# Teachers’ Strategies in Combating Diseases in Preschools’ Environments

**DOI:** 10.3390/children5090117

**Published:** 2018-08-30

**Authors:** Emmanuel Obeng-Gyasi, Melissa A. Weinstein, Jessica R. Hauser, Cecilia S. Obeng

**Affiliations:** 1 Department of Built Environment, North Carolina Agricultural and Technical State University, Greensboro, NC 27411, USA; eobenggyasi@ncat.edu; 2 Department of Health and Wellness, Curry College, Milton, MA 02186, USA; mweinstein@curry.edu; 3 Department of Exercise Science, Physical Education & Wellness, Tennessee Technological University, Cookeville, TN 38505, USA; jrhauser@tntech.edu; 4 Department of Applied Health Science, Indiana University School of Public Health, Bloomington, IN 47405, USA

**Keywords:** children, early care, diseases, vomiting, diarrhea

## Abstract

Scholarship on child health indicates that the early years of life are vital for all aspects of health and development. In particular, a solid health foundation predicts good life outcomes; therefore, systematic strategies for combating diseases are needed to ensure optimal health of young children in early care environments. This study examined strategies used by 48 preschool teachers from 10 pre-schools in the US Midwest in order to combat diseases. The following results emerged: Participants noted that children should not attend childcare if they are vomiting, have a fever or have diarrhea. They should be symptom-free for at least one day before returning to school. It is recommended that children be sent home if they have any disease deemed to have adverse effects on their health during the school day. Participants noted further that children must not share hats in their childcare environments to avoid transfer of head lice. Since no strategies were mentioned to help children cope with emotional distress and illness, it is recommended that teachers develop strategies that will address emotional childhood diseases in order to help all children.

## 1. Introduction

The early care environment is a particularly high-risk setting for exposing young children to infectious agents [1,2,3,4,5]. Given the close proximity of children to one another in the classroom, the sharing of toys and play equipment, as well as the lack of understanding of personal health behaviors, there is increased opportunity for germs to spread rapidly between children and adults. In order to reduce transmission of illness within an early care setting, it is essential to implement health guidelines for both classroom teachers and parents to abide by [2,3]. Young children carry their own combination of bacterial and viral agents from their homes to share with their peers and teachers. Personal hygiene and sanitation behaviors of young children are lacking; when considering their lack of full development and weakened immune system, they are more prone to diseases found in preschool environments [2]. The population of young children entering early care settings has changed drastically over the last several decades, given the shift in workplace equality and mothers going back to work after their children are born [3]. Children are entering early care environments at younger ages and for longer durations, thereby increasing their exposure time to potential infection.

Transmission of bacterial, viral, and parasitic infections that are particularly common in early care settings are spread through the nose, mouth, and feces, by way of droplets landing in the air, on surfaces and by direct contact [2,6]. Equipment that targets sanitation and hygiene behaviors such as hand-washing and preparing snacks in the classroom have significant impact on the control of illness in out-of-home care settings, benefiting both children and staff [4]. Using these hygiene and sanitation equipment and behaviors has yielded a reduction in diarrheal illness among children as well as fewer reported days absent by teachers [4].

Transmission of respiratory infections is another concern for early care settings [7]. Respiratory viruses are spread by way of nasal secretions. Infected droplets may land on other children or staff through coughing, whether through the air or by direct contact with the sick individual [2]. As young children have poor sanitary behaviors, they commonly do not cover or wipe their mouths of saliva or other fluid while coughing or after coughing. Communal surfaces may also be a hot spot for the collection of germs in this manner. To reduce the spread of infections through nasal secretions, it is important to enact strategies requiring children and staff to consistently wash their hands, with increased hand washing stations, repetitive use, and disinfecting all surface areas, including tables and chairs, food preparation space, and play equipment [2].

In addition to respiratory infections, the common cold, the flu, and other highly contagious infections such as conjunctivitis are prevalent among young children and have the potential to spread through early care facilities [2]. Common symptoms of these childhood infections that should be alarming to both parents and early care educators include fever, cough, sore throat, vomiting, diarrhea, and rash. These symptoms may prompt parents to keep their children home from school or teachers to call parents and send children home upon arriving at school. A variety of policies are used that may or may not be strictly enforced. Differences in policies may involve severity of symptoms, such as grade of fever and intensity of cough, and duration of persisting symptoms. The specific guidelines for keeping children home while ill can severely impact the spread of an illness [2].

Sanitary practices for food preparation and cleaning environmental surfaces are all essential strategies for early care staff. Cleaning and disinfecting policies are continuously highlighted [2,8]. Environmental cleaning strategies such as vacuuming, sweeping, mopping, wiping down surfaces and play equipment, and using cleaning products on food preparation areas are disease prevention strategies and are required in preschool settings. Hand washing frequently with warm water and soap is another consistently suggested prevention effort [9]. Hand washing has been shown to be the most commonly used practice of cleanliness in preschool classrooms, followed by teaching children about proper ways of coughing and covering their mouths as well as having discussions about germs [6]. Hand washing is recommended before eating, drinking, direct contact with a sick individual, after diaper changing and using the bathroom, blowing one’s nose and coughing [6]. Additionally, school attendance policies for children with clear symptoms of illness, being alert of children’s behaviors at the onset of an illness and containment of illness/disease transmission process [7], are essential in preschool settings, as is the need for parents to have a plan when serious symptoms persist, and alternative care needs to be sought [2].

Out-of-home care increases children’s susceptibility to infections, highlighting the importance of prevention efforts to protect children in preschools [1,2,3,4,5]. Given the increased attendance rates, heightened health risks, and rapid transmission of infection among this population, it is essential to further investigate specific strategies and practices that have been put into place by preschool educators to gain knowledge about which strategies have been most successful. Learning more substantially about common illnesses in young children and how they transmit (or are transmitted) as well as effective methods in combating the spread of such germs will enable public health professionals to create and implement safe and useful health policies to reduce unhealthy habits and illness in preschool environments.

Little has been written on the strategies teachers use in combating disease in the preschools’ environment. Given the gap in knowledge, this study sought to determine the strategies teachers use in combating diseases in preschools environments in the Midwestern USA to provide a baseline for changes over time, identify associations with potential predictors, and to identify targets for intervention.

## 2. Materials and Methods

### 2.1. Population Sample

To test the hypothesis that there are disease-based challenges in preschool environments this study was initiated with the objective being to determine the strategies teachers use in combating diseases in the preschools environment. The study was approved by an institutional review board.

The population for the study consisted of 48 early care educators in a Midwestern (USA) state from 10 preschools. Inclusion in the study required that the teachers work in preschool environments and teach three to five year olds. Participants who teach three to five year olds were selected for this study in order to discover the strategies they employed in combating diseases in this age group. The other reason for targeting young children between the ages of three to five was that it is likely that the findings of the study would have the longest impact on the development of these young children as exposures to toxicants and other agents occurring in these settings could affect children during their life course [10].

### 2.2. Procedure

The questionnaire for the study was developed by a researcher (the last author on this manuscript) with over 15 years of experience in preschool health education research and teaching. The questionnaire was given to another experienced researcher with over 30 years’ experience in qualitative research to check for content appropriateness of the questions.

The questions were pilot-tested with 10 participants. The ten participants who completed pilot-tested questions were not added to this study because the pilot-tested questions contained one fewer question (“Do you give all parents all written policies and strategies used in your classroom?”) than the questionnaire used for this study. The instrument used in the study consisted of both closed-ended and open-ended questions. The closed-ended questionnaires dealt with the demographic information of participants. The open-ended questions were used as the main source of data for this study.

The instrument covered their age, education, and strategies that they employed in combating diseases in their preschools’ environment, whether they have strategies that are communicated orally and those that are written that are given to parents, and whether they gave all parents copies of the written strategies and also informed them about their practices, if any.

Prior to the research assistants going to the preschools, phone calls and word–of-mouth conversations were made by the researchers with the schools to get their consent. All schools approached consented and respondents participated voluntarily.

Questionnaires for the study were administered in 2012 by two paid graduate research assistants and the last author. The criterion for selecting schools to take part in this research was based on their close proximity to the university where the last author on this manuscript and the graduate assistants work and study; the close proximity allowed the graduates research assistants to walk to collect the data. Ten early care settings qualified, based on the above-mentioned criterion, to participate in the study.

### 2.3. Coding Data Analysis

The coding was done by the lead author and the last author and 8 qualitative research assistants. In order to gain consistency, triangulation was used in which two methods of coding were performed in this research: Constant Comparative Method and an aspect of Grounded Theory (Selective coding). All the coders compared categories of similar information [11] and all the categories identified were put under major themes [12]. Selective coding was used because the coders chose one category to be the main category, and all relating categories were discussed under that theme. The 8 research assistants were paired, and each group coded the data separately.

To confirm the credibility and validity of the research, the coders used peer debriefing and scrutiny, which helped the coders incorporate each other’s suggestions, allowing the coders to take fresh comments into consideration [13]. This procedure also allowed for the confirmation of the themes, comparing results across the coders strengthened the study’s internal validity and reliability. The coders re-checking identified themes in this manner also aided in the reduction of potential biases in the study and ensured the accuracy of the findings.

To further ensure credibility and validity the lead and last author went over the data to check whether all numbers and the categories identified by the coders were the same. The analysis involved percentage calculations based on the total number of participants. Analytical claims made were based on the qualitative analysis of the participants’ answers to the open-ended questions from the surveys.

## 3. Results

### 3.1. Demographic Indicators

Results from the study indicated that there were 46 women and 2 men with a mean age of 37. One participant had a doctorate degree while a majority of the participants (*n* = 18; 38%) had a bachelor’s degree. Some participants had lower levels of education. There were 34 teachers from private preschools and 10 from institutional preschools and 4 teachers from a Church preschool (see Table 1 for details about participant’s background and schools).

In this study the majority (*n* = 43; 90%) of teachers and (*n* = 9; 90%) of preschools indicated that they had written strategies in their preschools.

Note that participants were not told the definitions of fever or diarrhea before or during the study. From our data the following themes emerged regarding the strategies that participants had in combating diseases in their preschool’s environment. The three themes below appeared the most from our data:

1. Strategies on fever
2. Strategies on vomiting
3. Strategies on diarrhea

### 3.2. Fever

For a fever to classify as alarming in children younger than 36 months, a digital rectal thermometer must read a minimum of 100.4 °F [14]. On the contrary, a fever of this magnitude is infrequently related to teething [14]. If a child had a fever, it is important to be alert for additional symptoms, as fevers can be associated with a multitude of serious infections [14]. The primary causes of fevers in children have shifted with the increased and widespread use of immunizations; however, perceptions regarding how to manage a child’s fever among healthcare professionals, parents, and caregivers have yet to be updated [14,15,16].

In this research, a majority (*n* = 27; 56%) of the teachers and (*n* = 7; 70%) of preschools indicated that they had strategies in combating fever in their preschool’s environment. Children are sent home if they had a temperature of at least 100 °F and if their temperature was over 101 °F if the temperature is taken under the arm. In this study the majority of the preschool educators indicated that children need to stay at home for 24 h and should also be fever-free before returning to their preschool. The following excerpts from the transcripts support the above finding:

- “Fever over 100 go home until fever free for 24 h”
- “Fevers are reasons we will call parents and send children home with a pink slip saying they cannot return until they are symptom free for 24 h without meds or with a doctor’s note”
- “Fever is defined as a temperature of 100 °F or higher taken under the arm, 101 °F taken orally, or 102 taken rectally”
- “Child cannot come back until medication has been taken for 24 h and are free of fever for 24 h”
- “Must be free of fever for a full 24 h before returning to class”

### 3.3. Diarrhea

Diarrhea among infants and children is defined by three or more loose or watery stools within a 24 h period [17]. In the United States, diarrhea is among the leading illnesses for children younger than 5 years of age [17]. To prevent dehydration, which is often associated with diarrhea, a therapy known as oral rehydration has been used. This process has been recommended in two phases to prevent dehydration. These phases include the rehydration phase where electrolytes and water, given as oral rehydration solution, replace fluid losses. In addition, there is the maintenance phase, in which there is replacement of outgoing fluid and electrolyte losses in addition to adequate dietary intake [18,19]. More recent studies are looking into the effectiveness of probiotics to aid in the prevention of and recovery from diarrhea. However, researchers have found that using probiotics are dose and strain specific [20].

From participants’ written responses, we observed that some teachers (*n* = 17; 35%) had strategies in their preschools environment on diarrhea which represented (*n* = 6; 60%) of preschools in which they stated that the director would call a child’s parents to come and pick them up from school if the child used the bathroom three times with loose stools. Others were specific and stated that children should stay home for a full 24 h before returning to school. There were some centers that insisted on written notes indicating symptom-free diarrhea before children could go back to school. The excerpts below support the above-mentioned analytical claims:

- “Must go home if child has 3 loose stools in same day”
- “Sent home (and home for a day after symptoms clear) if 3 consecutive diarrheas”
- “Must be free of diarrhea for a full 24 h before returning to class”
- “A child must have written notes before coming to school”

### 3.4. Vomiting

Vomiting is an organized autonomic response inducing the ejection of gastric contents through the mouth [21]. Vomiting continues to be a frequent symptom among children, and commonly serves as a condition that takes them to the emergency room [22]. Vomiting in children younger than 5 years of age should not be taken lightly; it can be a serious health risk and oral rehydration therapy should be started immediately, similar to the management of diarrhea, to prevent dehydration [22]. 

Teachers (*n* = 11; 23%) had written strategies in their preschool’s environment clearly stating that vomiting students should not be admitted in their centers and that they should be free of vomiting before coming to school this represented (*n* = 5; 50%) of the schools. They also stated clearly that parents should call to inform the center of their child’s illness. Participants said while vomiting, children do not do well in the learning environment. Some centers’ strategies in combating diseases in their preschool’s environment covered assurance to parents and encouraged them to be understanding and supportive when their child is vomiting; the centers gave promises to keep children in the Center as healthy as possible. Five excerpts are cited in support of the above-mentioned claims.

- “Vomiting—two or more times in a 24 h period. Return after 24 h vomiting free”
- “Excessive vomiting, we will call parents and send children home with a pink slip saying they cannot return until they are symptom free for 24 h without meds or with a doctor’s note”
- “Vomiting two or more times in 24 h, keep them home and call us to let us know they are not coming to school”
- “Do not bring your child to school if she is vomiting. We will do everything we can to keep your child as healthy as possible”
- “Must stay home if they are vomiting”

### 3.5. Written and Oral Health Strategies in Combating Diseases in Their Preschools Environment

Out of the 48 teachers, 27 (56%) had all their strategies written or documented and 21 participants had theirs both written and presented orally. These teachers were coming from (*n* = 10; 100%) of schools. Concerning health strategies in combating diseases in their preschool’s classrooms on information given to parents, 43 teachers (90%) said they gave all parents all written health information, while 5 participants (10%) gave some of their written health information to parents. The above was coming from (*n* = 10; 100%) of preschools that participated.

Some of the health information that were given to parents included short-term illness information. Parents did not get information about staff physical checkups unless they asked about them. They also did not get hand-washing procedures if they did not ask for them.

A majority of the teachers *n* = 25 (52%) indicated that to reduce the risk of children getting head lice from their classmates during dramatic play, hats must not be shared. These teachers came from (*n* = 6; 60%) of preschools.

## 4. Discussion

From the above stated results and the transcripts of participants’ responses, we infer that the majority of the teachers (37) from (7) schools reported a one-component definition of health (the physical health). The participants neglected other health dimensions such as mental and social well-being. The World Health Organization, however, defines health as “a state of complete physical, mental and social well-being and not merely the absence of disease or infirmity” [23]. From the above definition, participants will need to have strategies that cover emotional health diseases as well as the social well-being of the children they care for, for the betterment of the children’s overall health.

Concerning illness, participants indicated that they sent children home, especially if the children showed symptoms of diarrhea and vomiting. It is important for care-givers to note that a complete history of the children’s health should be taken into consideration to enable them (the care-givers) to know if the symptoms are possibly coming from a serious bacterial or viral infection [22]. The burden should not fall on the teacher alone and a more comprehensive strategy to adequately assess children’s health needs would be most suitable.

The fever guidelines elucidated in McDougall and Harrison’s [16] work and that of the National Institute for Health and Care Excellence (2013) provide guidance for managing fever in children aged 5 and younger. This recommendation calls for: The removal of excess clothing in order to allow the child’s body to cool through radiation and convection; offering of cold drinks to rehydrate the child and promote cooling, giving antipyretics given that antipyretics have analgesic properties and may reduce metabolic demands, and avoiding tepid sponging or bathing the child in cold water as cooling skin may induce shivering and increase body temperature.

The above guidelines give parents and other caregivers the ability to manage a fever, and as Demir and Sekreter [15] note, fever is a common symptom among pediatric patients and one of the most common reasons for taking a child to the doctor.

The teachers in this study understood the dangers of fever but their limited views of it and the potential differential diagnosis, some of which may come about from injuries [24] producing similar symptoms indicates that more compressive training is needed in preschool environments. In addition, fever, though bad, may be beneficial to the developing body’s immune system, and medications such as antipyretics do not prevent febrile convulsions [13,14]. Thus a more comprehensive understanding of the pathogenesis of fever, as well as other disorders which produce fever-like symptoms, is key to preventing diseases in the preschool environment.

The findings highlight gaps in relevant definitions of health among early childhood educators and have implications for interventions that will ensure the optimal health of preschool-aged children.

Also, given that a majority of the participants seemed to have no strategies on emotional health diseases, it may be noted that helping children with emotional health problems may be problematic in the studied preschools’ environment. To rectify the above-stated problem, the study recommends strategies in combating diseases in their preschool’s environment to be holistic (to cover all diseases including emotional health diseases) in order to help improve child health. Furthermore, early care educators should get the necessary training that can help them recognize diseases beyond physical diseases in their settings.

In all, the appropriateness of the tools, processes, data collection and interpretation made this a strong qualitative study examining the perceptions of teachers, their potential gaps in knowledge, and possible ways to remedy it.

A limitation identified in the study is that although we made sure that all preschools that serve children five years and under could have taken part in this study, only schools with close proximity to the researchers’ university were selected; thus, the schools were selected based on location; we could have obtained different results if the research area was expanded to other geographical areas with preschools outside our focused area. Future study will therefore focus on broad geographical areas; we will also study emotional health diseases that affect early care settings.

## 5. Conclusions

In conclusion, though strategies exist in early care environments to fight diseases, the majority of teachers reported a one-component definition of health (physical health), and participants who talked about other components only discussed information that will prevent physical diseases. Teachers indicated that they sent children home, especially if the children showed symptoms of diarrhea and vomiting. This indicates that training is needed to provide a more comprehensive understanding of preventative strategies in preschool environments. Also, given that a majority of the participants may have had no strategies regarding improving emotional health, it is suggested that in preschool environments, teachers may need to receive training on how to address these issues.

## Figures and Tables

**Table 1:** Descriptive Profile of Participants (*n* = 48).

Characteristics	% (*n*)
**Sex**	
Males	4 (2)
Females	96 (46)
**Age**	
18–25	29 (14)
26–35	27 (13)
36–45	21 (10)
46–55	17 (8)
56–65	6 (3)
**Education**	
High school graduate	13 (6)
Some college	23 (11)
Associates Degree	13 (6)
Bachelor’s Degree	38 (18)
Master’s Degree	13 (6)
Doctoral Degree	2 (1)
**Preschools**	
Private School	70 (7)
Institutional Preschool	20 (2)
Church Preschool	10 (1)
